# SARS-CoV-2 IgG seropositivity in a cohort of 449 non-hospitalized individuals during Spanish COVID-19 lockdown

**DOI:** 10.1038/s41598-021-00990-4

**Published:** 2021-11-03

**Authors:** Patricia Torres Martínez, Paula Diaque García, María Rubio Salas, Raquel Rodríguez Sánchez, José Avendaño-Ortíz, Sandra Guerrero-Monjo, Felipe García, Miguel Ángel Llamas, Eduardo López-Collazo, Paula Saz-Leal, Carlos del Fresno

**Affiliations:** 1 EMPIREO Diagnóstico Molecular, Madrid, Spain; 2 LABIANA Pharmaceuticals SLU, Barcelona, Spain; 3 The Innate Immune Response Group, La Paz University Hospital Institute for Health Research (IdiPAZ), Madrid, Spain; 4 CIBER of Respiratory Diseases (CIBERES), Madrid, Spain; 5 Inmunotek S.L, Alcalá de Henares, Madrid, Spain

**Keywords:** Viral infection, Epidemiology

## Abstract

The Coronavirus Disease of 2019 (COVID-19) pandemic caused by SARS-CoV-2 led the Spanish government to impose a national lockdown in an attempt to control the spread of the infection. Mobility restrictions and the requirement of a medical prescription for serological testing for COVID-19 were included among the control measures. Under this scenario, between April 15th and June 15th, 2020, we performed an observational study including 449 individuals allowed to be tested according to the governmental restrictions, i.e. fulfilling the following prescription requirements: manifestation of COVID-19-compatible symptoms, contact with a confirmed COVID-19 patient, or employment as an essential worker, including health care workers, firefighters and public safety personnel such as police. Importantly, a relevant feature of the studied cohort was that none of the participants had been hospitalized. We analyzed SARS-CoV-2 IgG seropositivity in this specific cohort, uncovering intrinsic features of great demographic interest. The overall rate of IgG seropositivity was 33.69% (95% CI: 29.27–38.21). This frequency was comparable among the different participant occupations. A RT-PCR positive test, contact with a household member previously tested positive and the presence of COVID-19-compatible symptoms were positively associated with IgG + results. Among these symptoms, ageusia/anosmia was positively and independently associated with SARS-CoV-2 IgG seropositivity, while odynophagia was inversely associated. However, fever, ageusia/anosmia and asthenia were the most frequent symptoms described by IgG + subjects. Therefore, our data illustrate how specific cohorts display particular characteristics that should be taken into account when studying population-wide SARS-CoV-2 seroprevalence and key defining symptoms of COVID-19.

## Introduction

Humankind is facing one of the most serious challenges in recent times due to the pandemic caused by SARS-CoV-2. This virus was identified early in January 2020 as the aetiologic agent for a pneumonia outbreak detected in Wuhan city, China, in December 2019. The resulting coronavirus disease 2019 (COVID-19) has quickly spread worldwide and was declared a global pandemic by the World Health Organization (WHO) in March 2020^1^, only three months after the first cases were detected.

Since its emergence, the scientific community has struggled to collect data from affected patients to better understand the pathophysiology of this infection. Notably, most infected individuals are asymptomatic^2^, and indeed, this is a critical feature to explain the rapid dissemination of SARS-CoV-2^3^. In symptomatic patients, clinical manifestations of COVID-19 range from mild to moderate upper respiratory tract illness, leading to either recovery or severe pneumonia and eventual death^4,5^. Fever, dry cough, dyspnea, loss of taste (ageusia) and/or smell (anosmia) and myalgia are among the most common symptoms^6–8^. However, as previously stated, most of the infected population does not present with symptoms or if they do, symptoms are not severe enough to seek for clinical treatment. These particular characteristics of COVID-19 have made it difficult to accurately ascertain the actual rate of SARS-CoV-2 infection and create a portfolio of common clinical features.

National governments are attempting to screen their populations in an unbiased manner to identify and isolate infected people and to determine the actual rate of infection^9^. This information could be informative, as under a scenario of wide-spread transmission of the virus, herd immunity could be achieved, which could help to control community-wide infection^10^. In this sense, the Spanish Ministry of Health performed an extremely large seroprevalence study called the ENE-COVID, analysing the presence of anti-SARS-CoV-2-specific IgG nationwide in more than 60,000 people chosen at random^11^. Seroprevalence for the entire country was determined to range from 3.7% to 6.2% in the first phase of the study, depending on the technique used for testing, which included either immunochromatographic or chemiluminescent immunoassay^11^. Interestingly, the seroprevalence distribution was not homogenous throughout the country, with some provinces showing a higher number of seropositive subjects for anti-SARS-CoV-2 IgG. For example, Madrid Province reported a seroprevalence between 11.3% and 11.5%^11^.

Seroprevalence studies have also been performed in the context of health care workers (HCWs), as they are the first line of defence against infection. These studies showed that SARS-CoV-2 IgG seropositivity was slightly higher in high-risk exposure personnel, with rates ranging from 9.3%^12^ to 10.3%^13^ in two studies performed in Catalonia (Spain), where the regional seroprevalence according to the national study ranged between 6.8% and 7.0%^11^. Therefore, the study of particular populations provides unique information about the impact of SARS-CoV-2 infection considering specific demographic features and reveals clues to better understand the pathophysiology of COVID-19.

In this study, we evaluated the association of demographics and clinical parameters with SARS-CoV-2 IgG seropositivity in Spain during the lockdown imposed by the Government between March 15th and June 21st, 2020. To do so, we analysed serological samples from a large cohort of 449 subjects for the presence of IgG against the SARS-CoV-2. The inclusion criteria included essential work, reported COVID-19-compatible symptoms, or contact with a confirmed COVID-19 case, with no hospital support in any case. Individuals that fulfilled any of these criteria were exempted from mobility restrictions and could undergo serological testing during this time period. Clinical and natural history data were collected by means of an anamnestic questionnaire and univariate and multivariate analyses were performed to identify specific features of this cohort. The analysis of this population and their intrinsic features provides valuable information about the rate of SARS-CoV-2 infection in a non-hospitalized cohort during the national COVID-19 lockdown. As indicated, the cohort included essential workers such as healthcare professionals, allowing comparative analysis of this key subpopulation.

## Results

### Baseline characteristics

**Figure 1:**
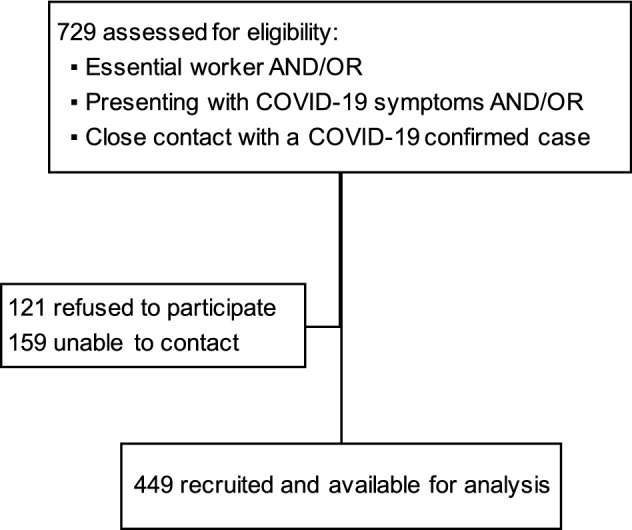
Flow diagram for inclusion in this study.

Initially, 729 eligible individuals met the inclusion criteria, and a total of 449 were included in the study after excluding 121 subjects who declined to participate and 159 who could not be contacted (Fig. 1). Among the participants, 55.68% were male and 44.32% female (Table 1). The mean age of the participants was 45.74 years (95% CI: 44.38–47.07). Sixteen percent declared themselves smokers and 22.94% reported having comorbidities (Table 1). Regarding occupation, 9.58%, 4.01% and 6.46% were healthcare workers (HCWs), firefighters and public safety workers, respectively, all of them were considered as essential workers during the Spanish lockdown. The rest of the individuals were not employed in these specific professions. As stated previously, due to mobility restrictions imposed during the lockdown, all the subjects resided in the Madrid province, with 33.18% of them in Madrid city.

**Table 1:** Baseline characteristics of study participants.

		n	%
Total		449	100%
Sex	Male	250	55.68%
	Female	199	44.32%
Age (years)	< 20	14	3.12%
	20–29	38	8.46%
	30–39	111	24.72%
	40–49	113	25.17%
	50–59	88	19.60%
	60–69	64	14.25%
	≥ 70	21	4.67%
Smoker	No	375	83.52%
	Yes	74	16.48%
Comorbidities^1^	No	346	77.06%
	Yes	103	22.94%
Occupation	Others^2^	359	76.96%
	Healthcare workers	43	9.58%
	Firefighters	18	4.01%
	Police/Public safety	29	6.46%
Municipality	Madrid area	300	66.82%
	Madrid city	149	33.18%
PCR status	Never done	403	89.76%
	Negative	33	7.35%
	Positive	13	2.89%
Contact with confirmed case	No contact	170	37.86%
	Household member	135	30.07%
	Non-cohabitating contact	144	32.07%
Symptoms compatible with COVID-19^3^	No	137	30.51%
	Yes	312	69.49%

^1^Comorbidities include: vascular system and digestive tract diseases, hypothyroidism, epilepsy, diabetes, chronic respiratory disease (asthma, allergy, COPD), cancers and autoimmune disorders.

^2^Those patients that are not among the other three specific professions.

^3^Fever, headache, cough, odynophagia, asthenia, myalgia, ageusia, anosmia, dyspnea, gastrointestinal symptoms, cutaneous manifestations and/or pneumonia diagnosis.

Forty-six participants (10.24%) had undergone previous testing by PCR, with 28.26% receiving positive results. Sixty-two percent of the participants reported contact with a confirmed COVID-19 patient who was either a household member (48.39%; 30.07% of the total) or a non-cohabitating person (51.61%; 32.07% of the total). In total, 69.49% of the included individuals reported COVID-19 compatible symptoms, which was also an inclusion criterion for the study (Table 1). The average time from the resolution of symptoms to testing was 42.56 days (95% CI: 39.90–45.23).

Notably, despite the manifestation of symptoms, none of the participants were hospitalized, which was a specific feature of the studied population.

### Frequency of IgG antibodies against SARS-CoV-2. Univariate analysis

First, we performed a univariate statistical analysis to assess the association between demographic/natural history characteristics and SARS-CoV-2 seropositivity. One hundred fifty-one participants were seropositive for IgG against SARS-CoV-2, corresponding to a prevalence of 33.69% (95% CI: 29.27–38.21). Sex, age, the presence of comorbidities and the location of residence were not associated with seropositivity (Table 2). However, the odds of being seropositive were lower in smokers (OR: 0.49, 95% CI 0.27–0.89) (Table 2). Notably, regarding occupation, no significant differences in IgG seropositivity were found among any of the specific occupations or overall essential professions and non-essential professions (Table 2). The fact that employment as an essential worker was a criterion for serological COVID-19 testing could introduce a bias in our results, as these participants did not need to declare COVID-19-compatible symptoms or contact with a confirmed COVID-19 patient. To address this concern, we analysed the two other inclusion criteria in our cohort of essential workers. This analysis indicated that the frequency of COVID-19-compatible symptoms was equivalent between essential and non-essential workers (Supp. Table 1). Furthermore, essential workers reported a higher frequency of contacts with a confirmed COVID-19 patient than non-essential workers (Suppl. Table 1), although there was no influence on the IgG seropositivity rate. These data suggest that the inclusion of essential workers did not introduce a bias in our analyses.

**Table 2:** SARS-CoV-2 IgG seropositivity by general characteristics.

		IgG Seropositivity			Univariate analysis			Multivariate analysis		
		%	(95% CI)		OR	95% CI	P-value	OR	95% CI	P-value^3^
Sex	Male	32.40	(26.64–38.58)		1		0.548^1^	1		
	Female	38.18	(28.56–42.24)		1.13	0.76 to 1.68		1.11	0.64 to 1.92	0.704
Age (years)	< 20	42.86	(17.66–71-14)		1		0.298^2^	1		
	20–29	34.21	(19.63–53.65)		0.69	0.20 to 2.43		0.67	0.14 to 3.04	0.603
	30–39	24.32	(16.68–33.38)		0.43	0.14 to 1.35		0.30	0.75 to 1.19	0.087
	40–49	33.63	(25.01–43.12)		0.68	0.22 to 2.09		0.46	0.12 to 1.81	0.268
	50–59	36.36	(26.31–47.31)		0.76	0.24 to 2.39		0.62	0.15 to 2.45	0.491
	60–69	42.19	(29.94–55.18)		0.97	0.30 to 3.13		0.91	0.23 to 3.66	0.895
	≥ 70	33.33	(14.59–56.97)		0.67	0.17 to 2.69		0.53	0.09 to 2.93	0.464
Smoker	No	36.00	(31.14–41.09)		1		0.021^1^	1		
	Yes	21.62	(12.89–32.72)		0.49	0.27 to 0.89		0.60	0.26 to 1.34	0.213
Comorbidities	No	32.08	(27.19–37.28)		1		0.235^1^	1		
	Yes	38.83	(29.39–48.94)		1.34	0.85 to 2.12		1.30	0.67 to 2.52	0.442
Occupation	Others	35.38	(30.43–40.57)		1		0.406^2^	1		
	Healthcare workers	25.58	(13.52–41.17)		0.63	0.31 to 1.29		0.59	0.22 to 1.63	0.311
	Firefighters	33.33	(13.34–59.01)		0.91	0.33 to 2.49		1.47	0.33 to 6.65	0.614
	Police/Public safety	24.14	(10.30–43.54)		0.58	0.24 to 1.40		0.38	0.94 to 1.57	0.182
Municipality	Madrid area	32.89	(25.42–41.05)		1		0.833^1^	1		
	Madrid city	34.00	(28.65–39.67)		1.05	0.69 to 1.59		1.09	0.60 to 1.96	0.782

^1^Fisher’s exact test.

^2^Chi-square test.

^3^Enter multivariate logistic regression.

OR: Odds Ratio. 95% CI: 95% Confidence Interval.

As expected, a significant association was found between self-reported positive PCR results and IgG seropositivity (92.31% IgG + among PCR + participants (OR: 29.98, 95% CI: 3.72–225.50)) (Table 3). Seropositivity was higher in those individuals who reported any contact with a confirmed COVID-19 patient (OR: 1.93, 95% CI: 1.26–2.94) than in those without contact. Among those with contact, the frequency of IgG seropositivity was higher among those for whom the contact was a household member (48.15%, OR: 2.83, 95% CI: 1.74–4.60) (Table 3).

**Table 3:** SARS-CoV-2 IgG seropositivity by self-reported clinical characteristics.

		IgG seropositivity		Univariate analysis			Multivariate analysis		
		%	(95% CI)	OR	95% CI	P-value	OR	95% CI	P-value^3^
PCR status	Never done	29.28	(24.48–33.99)	1		< 0.001^2^	1		
	Negative	42.42	(25.48–60.78)	1.78	0.86 to 3.67		1.52	0.53 to 4.33	0.432
	Positive	92.31	(63.97–99.81)	29.98	3.72 to 225.50		**21.97**	**1.91 to 252.92**	**0.013**
Contact with confirmed case	No contact	24.74	(18.42–31.89)	1		< 0.001^2^	1		
	Household member	48.15	(39.47–56.41)	2.83	1.74 to 4.60		**4.26**	**2.19 to 8.30**	** < 0.001**
	Non-cohabitating	29.86	(22.53–38.04)	1.30	0.79 to 2.14		1.69	0.83 to 3.41	0.146
COVID-19 symptoms	No	14.60	(9.15–21.64)	1			1		
	Yes	41.99	(36.45–47.68)	4.29	2.54 to 7.26	< 0.001^1^	1.052	0.12 to 85.71	0.982
Type of symptoms	Ageusia/Anosmia	77.36	(68.21–84.92)	13.57	8.02 to 22.96	< 0.001^1^	**15.30**	**5.98 to 39.12**	** < 0.001**
	Pneumonia diagn.^$^	75.00	(47.62–92.73)	6.35	2.01 to 20.03	0.001^1^	1.84	0.32 to 10.48	0.492
	Cutaneous	63.33	(43.86–80.07)	3.76	1.74 to 8.12	< 0.001^1^	1.53	0.46 to 5.06	0.483
	Fever	47.51	(40.06–55.05)	2.83	1.89 to 4.23	< 0.001^1^	1.59	0.75 to 3.36	0.225
	GI symptoms	50.47	(40.63–60.23)	2.57	1.65 to 4.02	< 0.001^1^	1.44	0.69 to 3.01	0.325
	Asthenia	44.51	(36.97–52.24)	2.19	1.47 to 3.27	0.012^1^	1.52	0.67 to 3.45	0.311
	Dyspnea	47.06	(34.83–59.55)	1.96	1.16 to 3.30	0.004^1^	0.62	0.23 to 1.70	0.355
	Cough	42.67	(34.64–50.99)	1.84	1.22 to 2.78	0.008^1^	0.99	0.47 to 2.10	0.981
	Myalgia	42.47	(34.93–50.51)	1.78	1.18 to 2.68	0.018^1^	0.66	0.30 to 1.47	0.309
	Headache	40.46	(33.08–48.18)	1.64	1.10 to 2.44	0.735^1^	1.02	0.48 to 2.15	0.956
	Odynophagia	32.20	(23.90–41.43)	0.92	0.59 to 1.43	< 0.001^1^	**0.34**	**0.16 to 0.69**	**0.003**
Number of symptoms	0	14.60	(9.15–21.64)	1		< 0.001^2^	1		
	1–3	32.59	(24.78–41.19)	2.83	1.56 to 5.13		1.82	0.45 to 71.96	0.751
	4–6	38.79	(29.89–48.29)	3.71	2.03 to 6.78		1.55	0.10 to 25.34	0.760
	7–9	70.00	(55.39–82.14)	13.65	6.33 to 29.45		2.47	0.27 to 22.31	0.421
	≥ 10	63.64	(30.79–89.07)	10.24	2.74 to 38.21		**7.46**	**1.74 to 32.03**	**0.007**

^$^diagnosis.

^1^Fisher’s exact test.

^2^Chi-square test.

^3^Enter multivariate logistic regression. Variables significantly associated with seropositivity are highlighted in bold.

OR, Odds Ratio. 95% CI, 95% Confidence interval. GI, Gastrointestinal.

**Figure 2:**
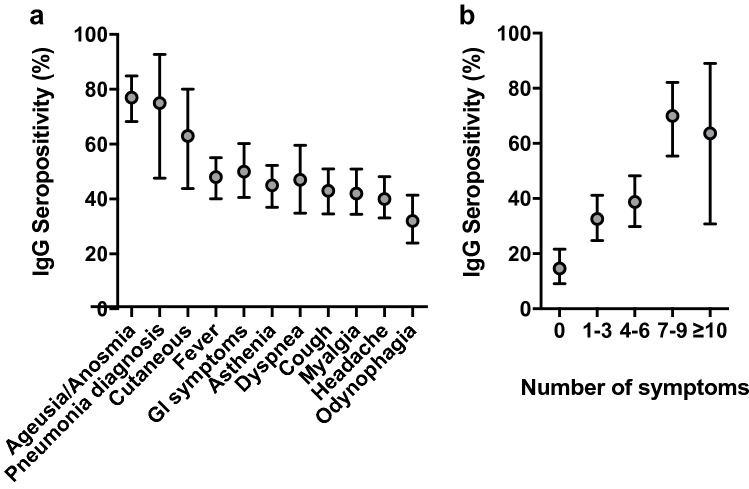
IgG seropositivity for SARS-CoV-2 by COVID-19 compatible symptoms and diagnosis. (**a**) IgG seropositivity associated with each of the reported symptoms or diagnosis. (**b**) IgG seropositivity grouped by the number of individual symptoms/diagnoses reported. Dots and vertical lines represent the mean and 95% confidence interval, respectively. GI, Gastrointestinal.

The odds of seropositivity were higher in participants who reported any COVID-19-compatible symptoms in the previous five and a half months (41.99% IgG + among participants with symptoms, 95% CI: 36.45–47.68 and OR: 4.29, 95% CI: 2.54–7.26 versus 14.60% IgG + among participants without symptoms, 95% CI: 9.15–21.64). The most frequent symptoms in IgG seropositive individuals were (in order): ageusia/anosmia (77.36%, OR: 13.57, 95% CI: 8.02–22.96), a pneumonia diagnosis (75.00%, OR: 6.35, 95% CI: 2.01–20.03) and cutaneous manifestations (63.33%, OR: 3.76, 95% CI: 1.74–8.12) (Table 3, Fig. 2a). The presence of symptoms was associated with IgG seropositivity (OR: 4.29, 95%: 2.54–7.26), even when ageusia/anosmia was excluded from the analysis (OR: 1.83, 95% CI: 1.03–3.24).

A larger number of symptoms was associated with a higher seropositivity rate (Table 3). Individuals reporting 7 to 9 or more than 10 compatible symptoms had a seropositivity rate that was approximately two-fold higher than that in individuals with 1 to 6 symptoms (Table 3, Fig. 2b). Besides, the analysis of IgG seropositivity within age ranges revealed that only the subgroup aged less than 20 years had a considerably larger frequency of asymptomatic IgG positive individuals (Supp. Fig. 1).

**Figure 3:**
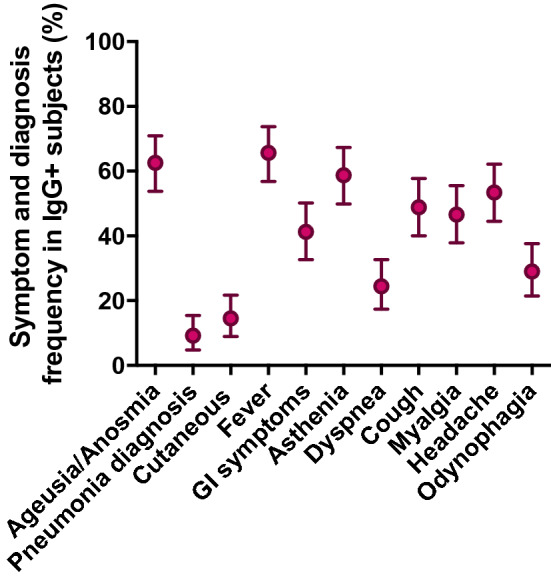
Symptom distribution among IgG positive subjects. Dots and vertical lines represent the mean and 95% confidence interval, respectively. GI, Gastrointestinal.

In an attempt to further explore the differential features of the cohort included in this work, the frequencies of the different symptoms were analysed among IgG-positive individuals (Fig. 3). Note that the symptoms are ordered according to the rate of IgG seropositivity, as shown in Fig. 2a. Fever, ageusia/anosmia and asthenia were the most frequent symptoms in descending order, whereas a pneumonia diagnosis and cutaneous manifestations were rare (Fig. 3). While these data sharply contrast with the overall associations observed between symptoms and IgG seropositivity (compare Fig. 2a versus Fig. 3), they reveal the differential features of the studied cohort, and the relevance of defining specific subpopulations to identify defining symptoms of COVID-19.

### Multivariate analysis

Once described the associations between SARS-CoV-2 IgG seropositivity and demographic and clinical parameters of the participants by univariate analysis, we performed two multivariate logistic regression models to test what variables were independently associated to IgG seropositivity.

First, a forward 6-stepwise selection model indicated that the smoker condition (OR: 0.44, 95% CI: 0.20–0.95), a positive RT-PCR result (OR: 25.12, 95% CI: 2.62–240.95), contact with a COVID-19-positive household member (OR: 3.72, 95% CI: 2.03–6.79) and having previous symptoms compatible with COVID-19 (OR: 2.49, 95% CI: 1.33–4.66), in particular, ageusia/anosmia (OR: 13.67, 95% CI: 7.32–25.53) and odynophagia (OR: 0.36, 95% CI: 0.19–0.66) were independently associated to IgG seropositivity against SARS-CoV-2 (Supp. Table 2).

Results from an enter logistic regression model in which all variables in a block were entered in a single step concurred in the association between IgG seropositivity and a positive RT-PCR result (OR: 21.97, 95% CI: 1.91–252.92), contact with a COVID-19 confirmed household member (OR: 4.26, 95% CI: 2.19–8.30), development of ageusia/anosmia (OR: 15.30, 95% CI: 5.98–39.12) or odynophagia (OR: 0.34, 95% CI: 0.16–0.69), along with showing 10 or more COVID-19-related symptoms (OR: 7.45, 95% CI: 1.74–32.03).

## Discussion

Between March 15th and June 21st, 2020, the Spanish Government imposed a national lockdown in an attempt to control the COVID-19 epidemic, which was caused by the spread of SARS-CoV-2 throughout the national territory^14^. Mobility restriction was an exceptional implemented measure, initially limited to the inhabiting place and subsequently limited to the surrounding province. Only those considered essential personnel, such as firefighters, civil servants preserving public safety and healthcare workers (HCWs), could travel to attend their jobs. People showing mild to moderate symptoms were asked to self-isolate at home, allowing the availability of clinical facilities for severe cases. The same preventive measures were required for asymptomatic persons known to have been in contact with a person with COVID-19-compatible symptoms. Under this scenario, a nationwide, population-based seroepidemiological study performed between April 27th and May 11st 2020, showed that seropositivity against SARS-CoV-2 in Spain was approximately 5% in the first study phase^11^. Interestingly, seroprevalence was not uniform throughout the country, with some areas displaying higher IgG positivity rates, such as Madrid, which had an 11.5% seroprevalence rate^11^.

Under this scenario, we performed the current study, analysing SARS-CoV-2 IgG seropositivity in individuals who were allowed to test for COVID-19 according to the governmental restrictions. This meant people that reported COVID-19-compatible symptoms at least two weeks before testing, had contact with a confirmed COVID-19 patient or essential workers who maintained work during the lockdown. Importantly, our cohort was restricted to people than did not required hospitalization, as a differential key characteristic. Considering these criteria, in a large cohort of 449 individuals, we observed an IgG seropositivity rate of 33.69%. It is important to note that these inclusion criteria were imposed by the restrictions included in government order RD463/2020^14^, which represents a limitation of our study. Considering this, the 33.69% IgG seropositivity rate for SARS-CoV-2 is suggestive of a much higher incidence in our cohort than the overall Spanish rate and even the rate in Madrid Province^11^, where our study was performed considering mobility restrictions.

The high IgG + rate observed in our cohort was surely influenced by the inclusion criteria. However, this is the strength of the data provided herein. Our results illustrate how IgG seropositivity for SARS-CoV-2 increased in a population allowed to be tested, that did not require hospitalization, while occupation did not introduce a bias. To the best of our knowledge, no studies have analysed such a population without differentiating according to other factors, and our study factors were determined by the strict limitations imposed by the government. Therefore, the features included in our cohort are not included in wide population-based studies, such as those performed in Spain^11^ or Switzerland^15^, or in published studies addressing the seroprevalence against SARS-CoV-2 specifically in HCWs^12,13,16–19^.

Our data revealed an IgG seropositivity rate of 25.58% (95% IC: 13.52–41.17) among HCWs. Other studies focused on these professionals revealed rates ranging from 2%^17^, 7.5%^16,19^, and 10%^11–13^ to nearly 20%^18,20^. Our results are in the upper range and are backed by data from a large cohort of HCWs in a secondary teaching hospital in Madrid Province^21^. These results are consistent with a nearly two-fold higher risk among health-care workers in a Spanish national seroprevalence study, as the reference population for our cohort would be the IgG + prevalence in Madrid Province, which was 11.5%^11^. Similarly, in two independent seroprevalence studies performed in Barcelona Province, a nearly two-fold higher risk was described for HCWs, ranging between 9.3%^12^ and 10.3%^13^ compared with 7% in the general province population^11^. Additionally, in Germany, a population-based seroprevalence study denoted a 0.94% of IgG seroprevalence in the North Rhine-Westphalia region^22^, while a 1.6% seroprevalence rate was reported among healthcare workers of the University Hospital Essen located in the same region^17^.

Interestingly, no significant differences in IgG seropositivity were found between HCWs in our cohort and other high-risk exposure professions, including firefighters and public safety personnel such as police, in line with other studies^23,24^. These data suggest that although much attention is being paid to clinical, front-line workers, other public employees have significantly higher positive rates than the general population, and they should receive the same personal protection and training as HCWs to protect themselves from coronavirus infection. Indeed, all these key professions had the same IgG seropositivity rate as those with COVID-19-compatible symptoms and those with contact with a confirmed patient, which were the other two inclusion criteria for our cohort.

A relevant factor to explain the high IgG seropositivity against SARS-CoV-2 in the studied cohort could be the controlled period of time spent between the resolution the symptoms and testing. One of the abovementioned studies was performed in HCWs exposed to COVID-19 patients; these HCWs were required to quarantine for 14 days, after which seroprevalence was analysed^20^. Interestingly, the frequency of IgG-positive tests was highest among studies performed in HCWs (17.14%)^20^. It could be suggested that this high rate was due to exposure to confirmed patients, with enough time to develop a robust humoral IgG response^25^. In fact, plasma from COVID-19 convalescent patients which is used as a potential treatment for infection, had significantly higher anti-SARS-CoV-2 titers when the time from symptom onset to plasma donation was more than 42 days^26^. For the symptomatic subjects in our cohort, at least 14 days after the end of symptoms was required before testing, with an average duration of more than 40 days. Therefore, the time of suspected contact with the virus should also be considered and detailed in an attempt to achieve a better interpretation of the seroprevalence data from diverse cohorts.

The important value of our data is to provide information about anti-SARS-CoV-2 IgG seropositivity in a cohort where most of the subjects had COVID-19-compatible symptoms, but none of them required hospitalization. These data are relevant for the design of studies regarding herd immunity^27^. Several nationwide studies reported low seroprevalence rates^11,15,28^, which promoted pessimistic perspectives among experts regarding the achievement of herd immunity due to natural infection^29^, although large vaccination campaigns should be game-changing factors in this sense. Notably, the seropositivity observed in our cohort indicates that the frequency of SARS-CoV-2 infection may be quite heterogeneous between populations, and heterogeneity impacts herd immunity, changing the percentage of seropositive people required to achieve herd immunity^10^. Therefore, large population-based studies on herd immunity should consider this heterogeneity to avoid the over- or under-representation of certain populations with IgG-specific positivity.

Although the test we used in this work detected both IgM and IgG against SARS-CoV-2, we based our study exclusively on IgG. The main reason was the inclusion criteria of COVID-19-compatible symptoms at least 14 days before testing to complete the quarantine period established by the sanitary authorities. In line with this rationale, only 2% of the individuals included in our cohort were IgM + . Indeed, IgG detection is the basis for most if not all population-based seroprevalence studies^11,15,28^. However, it does not preclude the usefulness of IgM determination for early infections together with PCR as the gold standard, showing a great concordance with IgG in the long term^30^. In agreement with other works^11^, we observed an association between IgG + seropositivity in individuals who self-reported a previous positive PCR test. However, frequency was not 100% and in fact, this result was consistent in essentially all the analysed studies^31,32^. False positive or negative results could explain this supposed discrepancy to some extent. However, the generation of T-cell-based cellular immunity could also be an underlying mechanism^33^.

The generation of immunogenic CD4^+^ and CD8^+^ responses against SARS-CoV-2 antigens that correlate with the presence of specific antibodies against the virus has been described^34,35^. However, up to 35% of unexposed donors (without detectable anti-SARS-CoV-2 specific antibodies) had SARS-CoV-2-reactive CD4^+^ T cells, while 83% of antibody-harbouring patients displayed comparable cellular responses^36^. Interestingly, SARS-CoV-2-responsive CD4^+^ T cells from unexposed donors also responded to similar epitopes present in endemic coronaviruses 229E and OC43^36^. Therefore, cross-reactive T cells might be the basis for cellular activation in the absence of a humoral response, although the dynamics of this cross-reactivity are not yet understood. As a T cell expansion is observed in mild versus severe COVID-19^37^, it is tempting to speculate that T cell immune responses are predominant in mild SARS-CoV-2 infections.

Cellular immunity mediated by T lymphocytes could also explain the presence of uninfected household members. Although IgG seropositivity was significantly increased in subjects who had contact with a confirmed SARS-CoV-2 infection patient^11^, nearly half of the individuals in our cohort were seronegative for coronavirus despite sharing a home with a COVID-19-positive person. Interestingly, only mild symptoms were reported in a cohort of non-hospitalized household contacts with SARS-CoV-2 infection^38^, further supporting the notion that cellular immunity triggered by mild infections could underlie the lack of seroconversion. Large studies comparing mild versus severe COVID-19 cases and analysing both cellular and humoral responses are required to fill this gap in knowledge.

An intriguing finding from our study is that smokers might had a lower rate of IgG seropositivity against SARS-CoV-2. There could be some plausible explanations for this effect, but they are currently only speculations. Explanations include a direct toxic effect of smoking on the virus, the impact on hACE2 expression as the viral entry gate or a pre-inflammatory status of the pulmonary tract, which helps to reduce the initial virus burden or even reduce the production of antibodies. In any case, a large observational study enrolling nearly 150,000 participants found that the rate of SARS-CoV-2 infection among current smokers was half that among non-smokers^39^, and this has also been described in smaller cohorts akin to ours^40,41^.

The presence of compatible symptoms as an inclusion criterion in our study allowed us to study the relationships between symptoms and seropositivity in the studied population. Notably, there was a positive correlation between the presence of symptoms and the anti-SARS-CoV-2 IgG positivity. This could suggest that the physical manifestation of COVID-19-compatible symptoms reinforces the development of antibodies against the virus^42^. However, there is also no unique, specific symptom to identify COVID-19, although this lack of specificity is attenuated by a combination of non-specific manifestations, as shown in our data and other studies^43,44^.

Along this line, it is interesting to note that the correlation of some symptoms with the anti-SARS-CoV-2 IgG seropositivity were much stronger than those of others. This supports the notion that COVID-19 manifests with some sort of symptomatic specificity, which might help to identify and isolate infected individuals in a potential scenario of a shortage of diagnostic tests. The development of ageusia/anosmia, cutaneous manifestations and diagnosed pneumonia were highly correlated with IgG seropositivity in the univariate analysis, with rates ranging between 60 and 80% in the presence of any of these symptoms, while ageusia/anosmia and odynophagia were the most associated in multivariate regression models, the latest, inversely correlated. Overall, these data would indicate that ageusia/anosmia is the best clinical indicator for the diagnosis of COVID-19^45,46^ in non-hospitalized people, while odynophagia could be a confounder factor of flu or common cold.

In sharp contrast, when considering the symptoms developed in the IgG + participants in the studied cohort, diagnosed pneumonia, cutaneous manifestations and even dyspnoea were among the less frequently presented symptoms. This fact signifies that depending on the intrinsic features of the participants, some symptoms are more or less indicative of COVID-19. The studied cohort in particular, comprised a population of high-risk exposure and symptomatic individuals that did not required hospitalization; therefore, they were unlikely to receive a clinical diagnosis of pneumonia or dyspnoea or develop cutaneous manifestations that required detection by skilled medical personnel. Consequently, the presence of ageusia/anosmia could be proposed as the most accurate indicative symptoms for COVID-19 in the general population without the need for medical assistance^47^. Its development could prompt self-isolation and to contact with health authorities.

In summary, the data presented illustrate IgG seropositivity against SARS-CoV-2 in a non-hospitalized population allowed to be tested for COVID-19 during the Spanish lockdown. The seropositivity was nearly two-fold higher than described in a population-based, nationwide study in the same geographic area. This relatively high seroprevalence was shared among different front-line public professionals such as healthcare workers, firefighters and public safety personnel. Importantly, the seropositivity among these professionals was comparable to that in participants with other occupations who either showed symptoms or co-habitated with a confirmed COVID-19 patient. The presence of COVID-19-related symptoms was positively correlated with IgG seropositivity. Among these symptoms, ageusia/anosmia positively associated with higher rates of IgG seropositivity for SARS-CoV-2, while odynophagia associated inversely. However, the most frequent symptoms among IgG + participants were fever, ageusia/anosmia and asthenia. Therefore, heterogeneity among populations should be considered when defining seroprevalence and key diagnostic symptoms.

## Methods

### Study design and participants

This study was performed at the laboratory facilities of Empireo Diagnóstico Molecular (www.empireo.es) in Madrid. The collection of serological samples was conducted since April 15th to June 15th, 2020. Participants were contacted by telephone between June 1st and 21st, 2020 to provide their clinical and natural history following an anamnestic questionnaire. This questionnaire included all the baseline characteristics recorded in Table 1. The study was approved by the ethical review committee of Comunidad de Madrid and all methods were carried out in accordance with relevant guidelines and regulations provided by the Consejería de Sanidad. Written informed consent was obtained from all study participants.

This study was performed during the COVID-19 lockdown period in Spain dictated by the Spanish government on March 14th, 2020, according to RD463/2020^14^. During this period, on April 14th, the government order SND/344/2020 established the requirement for a medical prescription for a diagnostic test for COVID-19^48^. We adopted a restrictive and ethical interpretation of this rule to identify potential participants. To be included in the study, participants needed to fulfil at least one of the following inclusion criteria: 1) symptoms compatible with COVID-19 (fever, headache, dry cough, odynophagia, asthenia, myalgia, ageusia, anosmia, dyspnoea, gastrointestinal (diarrhoea, vomiting), cutaneous manifestations (defined as urticaria, erythema or flaky dermatitis-like lesions) and/or a pneumonia diagnosis at least 14 days before testing to abide by the quarantine rules established by the sanitary authorities; 2) contact with a confirmed COVID-19 case based on a positive RT-PCR of such contact; and 3) employment as an essential worker (those allowed to keep working during the lockdown under strict accreditation by the institutional employer). It is important to stress that the government order SND/344/2020 defined these criteria as the only ones that allowed us to perform diagnostic testing for COVID-19^48^ at the time when the participants were recruited for this study. Furthermore, none of the participants required hospitalization as an additional inclusion criterion.

The SND/344/2020 order indicated that being an essential worker was criterion for serological COVID-19 testing, and this could introduce bias in our results. To address this concern, we analysed the frequency of the two other inclusion criteria (COVID-19-compatible symptoms or contact with a confirmed COVID-19 patient) in our cohort of essential workers (Supp. Fig. 1).

The exclusion criteria included refusal to participate, an unsigned informed consent form, or failure to obtain or complete the questionnaire for study records.

RD463/2020, implemented on March 14th imposed mobility restrictions for non-essential personnel, limiting movement between towns in the first stage and between provinces in the second stage. These restrictions constrained the territorial distribution of the included subjects mostly to Madrid city and province.

### Detection of SARS-CoV-2 antibodies

The test (REAL COVID19 Rapid test cassette; Durviz, Valencia, Spain; reference RPPCOV1925) was a lateral-flow immunochromatographic assay for qualitative detection and differentiation of IgG and IgM against SARS-CoV-2 proteins, which yields results in 10 min. The manufacturer reports 100% sensitivity (95% confidence interval (95% CI): 86%–100%) for IgG and 85% sensitivity (95% CI: 62.1%–96.8%) for IgM as well as 98% specificity (95% CI: 89.4%–99.9%) for IgG and 96% specificity (95% CI: 86.5%–99.5%) for IgM; RT-PCR is considered the gold standard. For these determinations, serum samples from 20 SARS-CoV-2 RT-PCR positive and 50 RT-PCR negative patients were analyzed. Following a method published elsewhere^49^, we adjusted of the obtained IgG seropositivity values, taking into account the intrinsic error of the kit. These adjustments are relevant to pooled data obtained in different studies. Considering the 100% sensitivity and 98% specificity, the performed adjustment involved a residual variation below 2%. No cross-reactivity in samples positive for influenza A or B, hepatitis B, syphilis or HIV was indicated. Serum was obtained from blood after centrifugation and assayed immediately according to the manufacturer’s instructions.

Due to the low seroprevalence of IgM in the cohort (approximately 2%), most likely due to the short duration after infection, and the time between the putative infection and the test performed as the first inclusion criteria, the results shown here are only based on only IgG.

### Statistical analysis

Seropositivity rates of antibodies against SARS-CoV-2 were calculated as proportions with 95% CIs (confidence intervals). Univariate analyses based on odds ratios (OR) were performed to evaluate factors associated with antibody seropositivity. ﻿We tested the associations between variables with the Fisher’s exact test or the chi-square test when two or more variables were analysed, respectively. For multivariate analysis two different approaches of multivariate regression models were performed, a forward stepwise selection method and an enter logistic regression model.

The analysis and handling of data were carried out using Microsoft Excel v16.16.08 (Microsoft, Redmond, WA), GraphPad Prism v6.0c (GraphPad software, San Diego, CA) and SPSS statistics v23 (IBM, Armonk, NY).

## Supplementary Information

Supplementary Information.
