# Supplementary material for: SARS-CoV-2 IgG seropositivity in a cohort of 449 non-hospitalized individuals during Spanish COVID-19 lockdown
Source: Sci Rep. 2021 Nov 3;11:21612. doi: 10.1038/s41598-021-00990-4 (PMC8566591; doi:10.1038/s41598-021-00990-4)
Supplement: Supplementary file 1 — Supplementary Information. [file 41598_2021_990_MOESM1_ESM.pdf]

SARS-CoV-2 IgG seropositivity in a cohort of 449 non-hospitalized individuals during Spanish COVID-19 lockdown.

Patricia Torres Martínez MD, Paula Diaque García, María Rubio Salas, Raquel Rodríguez Sánchez MD, José Avendaño-Ortíz PhD, Sandra Guerrero-Monjo PhD, Felipe García, Miguel Ángel Llamas PhD, Eduardo López-Collazo PhD, Paula Saz-Leal PhD, Carlos del Fresno PhD

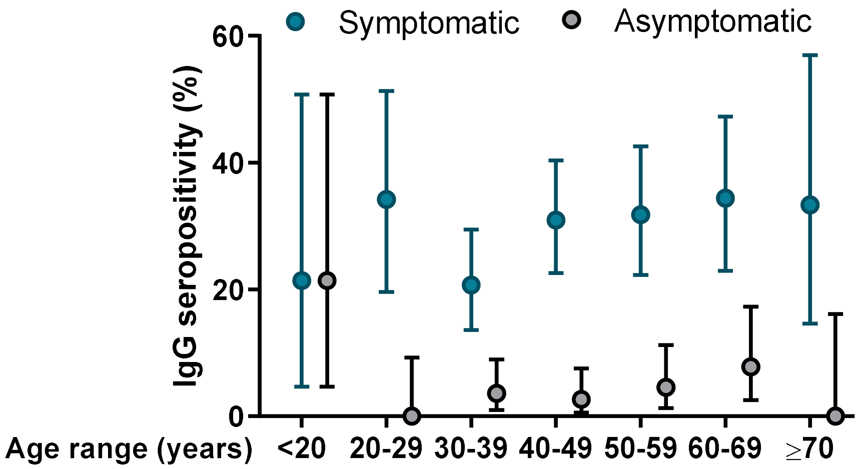

Symptomatic (%)	21.43	34.21	20.72	30.97	31.82	34.38	33.33
Asymptomatic (%)	21.43	0.00	3.60	2.65	4.55	7.81	0.00

Supplementary Figure 1. Symptomatology-dependent seropositivity of SARS-CoV-2 within age ranges. Dots represent IgG+ symptomatic (blue) and asymptomatic (grey) subjects at given age ranges. Dots and vertical lines represent frequency and 95% confidence interval, respectively. Frequencies are further detailed in the bottom table.

Supplementary Table 1. COVID-19 compatible symptoms and close contact of study participants by occupation.

		Frequency		Univariate analysis		
		%	(95% CI)	OR	95% CI	P-value
**Symptoms compatible with COVID-19[1]**	**Others[2]**	**70.47%**	(65.46-75.15)	1		0.366[3]
	**Healthcare workers**	**72.09%**	(56.33-84.67)	1.08	0.53 to 2.19	
	**Firefighters**	**66.67%**	(40.99-86.66)	0.84	0.31 to 2.29	
	**Police/Public safety**	**55.17%**	(35.69-73.55)	0.52	0.24 to 1.11	
**Any contact with confirmed case**	**Others[2]**	**57.10%**	(51.80-62.28)	1		<0.001[3]
	**Healthcare workers**	**79.07%**	(63.96-89.96)	2.83	1.32 to 6.09	
	**Firefighters**	**94.44%**	(72.71-99.86)	12.77	1.68 to 97.05	
	**Police/Public safety**	**72.41%**	(52.76-87.27)	1.97	0.85 to 4.57	

[1] Fever, headache, cough, odynophagia, asthenia, myalgia, ageusia, anosmia, dyspnea, , gastrointestinal symptoms cutaneous manifestations and/or pneumonia diagnosis.
[2] Those patients that are not among the other three specific professions.
[3] Chi-square test.
OR: Odds Ratio. 95% CI: 95% Confidence Interval.

Supplementary Table 2. Forward logistic regression model.

		Forward multivariate analysis		
		OR	95% CI	P-value
**Smoker**	**No**	1		
	**Yes**	**0.44**	**0.20 to 0.95**	**0.037**
**PCR estatus**	**Never done**	1		
	**Negative**	1.42	0.58 to 3.48	0.442
	**Positive**	**25.12**	**2.62 to 240.95**	**0.005**
**Contact with confirmed case**	**No contact**	1		
	**Household member**	**3.72**	**2.03 to 6.79**	**<0.001**
	**Non-cohabitating**	1.48	0.79 to 2.78	0.216
**COVID-19 Symptoms**	**No**	1		
	**Yes**	**2.49**	**1.33 to 4.66**	**0.004**
**Type of symptoms**	**Ageusia/Anosmia**	**13.67**	**7.32 to 25.53**	**<0.001**
	**Odynophagia**	**0.36**	**0.19 to 0.66**	**0.001**

OR: Odds Ratio. 95% CI: 95% Confidence Interval.
Variables significantly associated with seropositivity are highlighted in bold.
